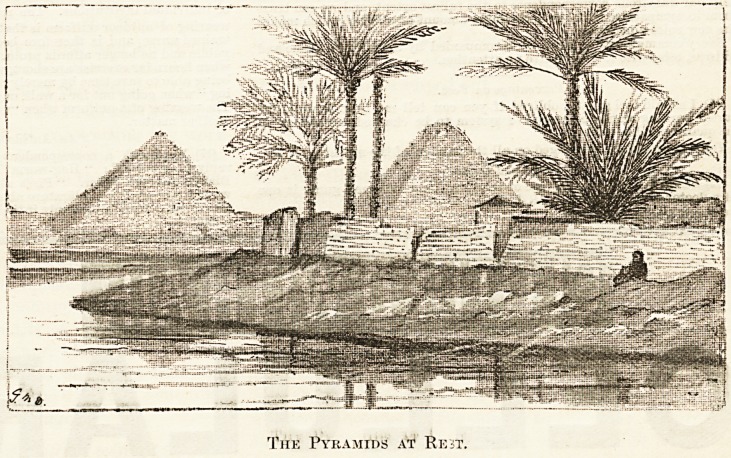# "The Hospital" Nursing Mirror

**Published:** 1899-04-08

**Authors:** 


"The Hospital" Nursing Mirror.

Being the Nursing Section of "The Hospital."

[Contributions for this Section of "The Hospital" should be addressed to the Editor, The Hospital, 28 & 29, Southampton Street, Strand, London, W.C., and should have the word "Nursing" plainly written in left-hand top corner of the envelope.]

### Notes on News from the Nursing World.

### THE WANTS OF ST THOMAS S HOSPITAL.

Among the urgent wants of St. Thomas's Hospital are, we learn from the annual report of Mr. J. G. Wainwright, the treasurer, increased dining and sitting room accommodation for nurses, the establishment of a complete laundry, and a more efficient system of bedding disinfection. Concerning these and other works, the report says: "It only needed the governors and friends of the hospital to fully realise how important it was that the hospital should be provided with such conveniences and appliances to secure the funds required." We hope that this is not an optimistic view. It ought not to be so. But the public are more quick to respond to an appeal for the erection of a new wing, which adds to the size of the structure, than they are to find money for such prosaic necessities as improvements in the laundry and disinfecting departments, or to minimise the discomforts of the hard-working nurses.

### A PLEASANT EVENING FOR THE NURSES OF BIRMINGHAM.

The Lord Mayor and Lady Mayoress of Birmingham have issued the following invitation to the whole of the nurses of the different hospitals and institutions of that city. "The Lord Mayor and Lady Mayoress of Birmingham (Alderman and Mrs. Beale) request the pleasure of Nurse ————'s company at the Council House on Tuesday Evening, April 25th, 1899." Accompanying the beautifully designed invitation card is the following printed circular: "As it is understood that some of the guests may be unable to accept the accompanying invitation for the whole of the evening, the Lord Mayor and Lady Mayoress will receive those who have to leave early, at 6.30! and those who cannot come till late, at 9.30. There will be an entertainment in the Council Chamber at 7 o'clock, and another at 10 o'clock; and the arrangements will be the same for both parts of the evening. If not able to stay for the whole evening, it is requested that the reply to the invitation may state whether it is accepted for the first or second part. It is requested that uniforms may be worn. Replies should be addressed to the Lady Mayoress, the Council House."

### MISS HONNOR MORTEN AND MUNICIPAL HOSPITALS.

We regret to observe that at the Socialist and Labour Conference at Leeds Miss Honnor Morten read a paper in which she advocated municipal hospitals, and maintained that they have many advantages over competitive voluntary hospitals in the matter of uniformity of system and economy of management. She urged, among other things, "that there was no reason why all our hospitals should not be under municipal management." But a great many even of the British public could adduce several cogent reasons against the change which Miss Morten advocates. To mention three only: Universal municipal hospitals would enormously increase the rates, which are already a terrible burden; would stereotype one system of management where diversity is needed; and would put a peremptory stop to a flow of charity which has done more than anything, except the multiplication of churches and religious agencies, to prove the reality of Christianity in this country.

### REMARKABLE DEATH-RATE AMONG GERMAN NURSES.

It is stated in a German missionary magazine that the average length of life among the Roman Catholic sisters who serve as nurses abroad is only 36 years. During the last quarter of a century no less than a third of these nurses died in the first five years of service, and three-fifths in the first 10 years. Sixty-three per cent. of them were the victims of lung disease. The causes to which the author of the statement attributes this are by no means inadequate. It appears that these unfortunate nurses enter at too early an age upon the work; that the labour is constant and heavy, without enough change; that the hours of sleep are too short; that there is too little recreation; that unsuitable covering is provided for the head; and that the clothing on the beds is insufficient. Here, of course, are the conditions that invite consumption, but the publicity given to them ought to go far to effect their removal. The idea of an English nurse's life in any country averaging no more than 36 years would deter from volunteering for work in our dependencies girls who have no fear of ordinary risks, but who might fitly shrink from being cut down in the flower of their womanhood.

### OF THE VICTORIAN ORDER.

Lady Aberdeen has quitted Canada, but she has left behind her an enduring monument of her own work in the Victorian Order of Nurses founded during her husband's Viceroyalty. There were many difficulties to contend with, much prejudice to overcome, and it was some time before the Order designed to work for Canada as the Queen's Nurses work for Great Britain took root downward in the hearts of the independent colonists. But the seedling lived, and the first report, indicating marked growth, was presented on March 10th to the annual meeting held at the Canadian Institute, Toronto. Miss Eastwood, the local lady superintendent, gave a review of the work, which began on March 12th, 1898. The Toronto staff consists of four trained nurses, who are receiving a course of instruction in district nursing. From time to time these are drafted on to other districts, and new probationers take their places. Miss McLeod, general lady superintendent, told the meeting of the branches established at Ottawa, Montreal, Toronto, and Halifax; of the cottage hospitals at Cape Breton, and at other places; of Lord Aberdeen's gift to equip another hospital for six months at New Richmond, Quebec; of the 25 nurses who have rendered heroic service in the Klondyke district, where they are still working. The troops under Colonel Evans have subscribed £70, and presented the Order with a cabin. One nurse, Miss Powell, walked 150 miles in the wake of the typhoid epidemic until she herself became prostrated by the disease. The medical men of Canada are now recognising the value of the nurses, Dr. Harley Smith declaring that any objection to the Order was now ancient history, whilst Dr. Temple remarked that it was a great boon to poor people.

### BIRMINGHAM AND MIDLAND COUNTIES TRAINING INSTITUTION FOR NURSES.

THE annual meeting of the governors of this institution was held last week at the Home, Birmingham, the Lord Mayor (Alderman Beale) presiding. The committee, in their report, stated that the work of the institution had been carried on with undiminished activity and usefulness during the year. It was, however, with profound regret that they recorded the serious loss which had befallen them in the death of the late lady superintendent, Mrs. Diamond, who for 23 years performed the duties of her responsible position with great ability and devotion to the interests of the institution. During the year 584 families had been attended, the cases including 24 of scarlet-fever, 19 of typhoid, and 53 of other infectious diseases. The committee continued to receive very gratifying reports of the conduct and general capabilities of the nurses. In the course of the year several of the nurses had been detained at the Home by illness, and had been most kindly attended by the honorary medical officers, Dr. Rickards, and Mr. Gilbert Barling. Twelve nurses had left the institution and two were now enjoying the benefits of the pension fund to the extent of £20 per year each. The number of nurses now on the staff is 73. The usual contribution of £150 had been made during the year to the District Nursing Society. The usual yearly bonus of £100 had been distributed amongst the nurses who had completed six years' service for the institution, and a sum of £100 had been placed to the credit of the sinking fund. In moving the adoption of the report, the Lord Mayor complimented the officers on its concise character. He said that the institution was on a sound commercial basis, and it was gratifying to know that basis was sure whatever happened when the lease of the present Home expired. At the same time, one did not forget that the institution was founded to supply a real want, and to a certain extent it was the parent of, and greatest contributor to the District Nursing Society, which they all desired to see extended. Mr. J. Courtney Lord seconded the motion, and said that the nurses trained in the institution were the best that could be found in Birmingham. In order to keep up the traditions of the institution they had determined to have their nurses now trained for three years, and had made arrangements whereby nurses who had been on the staff for some years could return to the hospital for a time and have the advantage of learning the new modes of treatment which science was almost every day bringing forward. The committee were reappointed, and Mr. H. F. Osler and Mr. R. F. Impy were reappointed respectively treasurer and hon. secretary. Votes of thanks were accorded to the honorary medical officers and officers for their services, and a vote of thanks to the Lord Mayor closed the meeting.

### CORNWALL NURSING ASSOCIATION.

THE Earl of Mount Edgcumbe presided at the annual meeting of Cornwall County Nursing Association recently held at Truro; there was a large and influential attendance. The work of the association continues to make good progress, and the nurses have been well employed and have rendered excellent service. The village nurses, trained by the association free of cost to themselves, have been started with a view to meet the want of trained nursing in scattered rural districts, where it is impossible to raise the sum required to maintain a Queen's nurse. The minimum time required for training village nurses is six months, but the Executive Committee have decided in some instances to allow twelve months instead, so as to better qualify them for their work. These nurse. are required to pass the examination of the London Obstetrical Society, and to obtain its certificates Arrangements have been made to give the village nurses six months' additional training in the South Devon and East Cornwall Hospital, Plymouth, and the Royal Cornwall Infirmary, Truro, subject to certain conditions. The association has now altogether expended £229 on the training of nurses, and has received a grant of £100 from the Cornwall Technical Education Committee. The county superintendent (Miss Michie) has assisted the nursing work a great deal by inspecting and lecturing, and the Queen's Institute has awarded her its silver badge in recognition of her services. The association cordially invite non-affiliated town and country districts throughout the county to join them, because union is strength. Their central fund is not strong, and the committee appeal earnestly to all who have the welfare of the sick and suffering at heart to come forward and help them to carry on the good work. It is their aim to establish a nurse for the sick poor in every parish or group of parishes throughout the whole county.

### LEGACIES TO NURSES.

THE idea that nurses are more likely than most persons to be left large sums of money may, perhaps, derive encouragement from the case of Miss Amelia Edwards, who, thanks to a bequest from the late Mr. W. S. Broadwood, is now the possessor of the handsome sum of £30,000, a mansion with stables well stocked with horses and carriages, and freehold land at Malvern and Henley Castle. The remarkable esteem entertained by Mr. Broadwood for the lady who nursed him is further shown in the fact that he also left her all his private papers and correspondence, "without any liability on her part to produce them to anyone." But clearly this is an absolutely exceptional occurrence. Now and again a nurse receives from a grateful patient a substantial acknowledgement of her services in addition to her fees. These, however, are only the exceptions which prove the rule—namely, that nurses are no more than the members of any other profession the favourites of fortune.

### LADY HENRY SOMERSET ON THE NECESSITY FOR POLICE MATRONS.

THERE can be no doubt that Lady Henry Somerset rightly insists upon the urgent need of police matrons south of the Tweed. Lady Henry points out that the system has been at work in Glasgow for more than a quarter of a century, "where two or more police matrons are on duty at the central station all night, and one at each of the six district stations." It is also in operation at Edinburgh, and the excellent results are fully admitted. Lady Henry contends, with unquestionable force, that to allow matronly supervision is necessary for workhouse and infirmary, but to deny that it is necessary for a police inmate, "is to assume a principle which is altogether false—that any one crime unsexes the offender." But if there are to be police matrons, it is indispensable that they should possess medical and nursing knowledge, so as to enable them to distinguish between women who are brought into police stations the victims of drink and women who, as sometimes happens, are suffering from illness.

### AT RANGOON.

There is not much interest taken in the affairs of the Rangoon Municipal Hospital in this country, and a few months ago a good many people were puzzled by a report of the "strike" of nurses at this institution. Yet the hospital is a large place with wards for Europeans and natives, for men and women, as well as for contagious and moribund cases. There is undoubtedly a considerable amount of trouble with regard to the nurses. There are no proper quarters for them in the building, and the pay is too small to provide them elsewhere. The Superintendent of the hospital sent a letter on the subject to the President of the Rangoon Municipality in which he stated that although much had been done within the past few months by the direction of an English matron and two English sisters, yet there still remained a good deal to remedy. He recommended that there should be a resident trained matron at a salary of not less than Rs.200; that the salaries of the nurses should be raised; and that allowances should be made for residence and rations. He also thought that the hospital would benefit by the appointment of six attendants who would prove helpful in the wards. The matter has been referred to a special sub-committee for report.

### SUPPLEMENTARY TRAINING.

A week or two ago we hinted that the Irish Guardians who compassionated the case of the worthy but non-certificated nurse held the remedy in their own hands—they could send her to a training school to complete the necessary term of instruction, and thereby earn the parchment entitling her to serve under the regulations of the Local Government Board. In a letter from the Irish Local Government Board to the New Ross Board of Guardians, it is stated that the Local Government Board would be willing to approve of any reasonable arrangement for permitting existing workhouse nurses (with the approval of the workhouse medical-officer) to obtain leave of absence with a view to their becoming qualified as trained nurses. It is not contemplated that such an arrangement should apply to nurses to be appointed in future, but only to those nurses who are at present in the service of the guardians. If leave of absence is given to a nurse for the purpose, an efficient substitute will, we presume, be engaged by the guardians for the period during which the nurse is absent.

### DISTRICT NURSING AT TORQUAY.

The sooner the mass of the public awakens to its responsibilities and takes an active part in the support of such national charities as are represented by hospitals and nurses the better. Smugly and with self-complacency the majority of those who are happily as far removed from poverty as they are from opulence stand aloof. At most they are content to patronise concerts and other gaieties promoted to keep alive institutions essential to the well-being of society, and having obtained an excellent entertainment at a cheap rate plume themselves on having discharged their charitable obligations handsomely. Take, for instance, the report of the Torquay Nursing Association presented at the recent annual meeting. It is therein stated that there is work for ten district nurses in the town, and that only six are at present employed; that the expenditure is £400 a year, and that the subscriptions reach a meagre £190; that but for the donation of £123—the result of the Lady Mayoress's ball—the accounts would be hopelessly in arrears. Nor is this an isolated instance; the same tale is heard everywhere. The rich contribute their gold; the poor their pence, in addition to many a kindly deed. Why should the multitudes who stand between these two extremes withhold their silver?

### WOMEN'S TOTAL ABSTINENCE UNION.

The importance of temperance can never be overrated, and we are, therefore, pleased to give whatever encouragement is in our power to such societies as tend to promote habits of self-restraint. Mr. and Mrs. Allen recently lent their drawing-room at Woodside Terrace, Glasgow, to the Nurses' Branch of the Women's Total Abstinence Union. A large number assembled, and enjoyed excellent addresses from Dr. Reid and the Bishop of Glasgow. Amongst those present were Lady Bell, Lady Gairdner, Mrs. Kerr, and Dr. Henderson. Many of the nurses present joined the League.

### SHORT ITEMS.

Some of our readers may remember that Nurses Nutt, Clarke, and Powell, of Guy's, accompanied Major Lugard's expedition to West Africa. They have well sustained the reputation of their training school, and Dr. Poole attributes to their skilled attention the recent recoveries of his patients from Blackwater Fever.—In chronicling the changes that have lately taken place amongst nurses past and present of St. Thomas's, we note that Miss Wrigley, for many years the casualty and out-patient sister, is shortly leaving to take up her new work as Matron to the Cheltenham Hospital. Much regret is expressed on all sides, and a photograph of the out-patient staff has been taken as a souvenir. The Theatre Sister, Miss Froude, has been appointed Sister Elizabeth in the place of Miss Herbert, who is leaving; whilst Miss Innes becomes Theatre Sister. Miss Easton, who has recently been appointed Matron of the Reading Hospital, is a St. Thomas's nurse, and has proved her worth as matron of the Waterloo Road Hospital for Sick Children and Women.—Part I. of *The Art Portfolio*, which has just been issued by Messrs. Simpkin, Marshall, Hamilton, Kent, and Co., is full of promise. It consists of four admirable photogravure reproductions of well-known paintings, unencumbered by letterpress. The copy of Frederick Walker's "The Harbour of Refuge" is alone fully worth the modest shilling which is the price of the portfolio.—Mrs. Wadham's exertions to collect £800 to establish and furnish a nurses' home for the Barrow District Nurses' Association have been so successful that only £160 more are needed.—Among the saved from the wreck of the "Stella" was Miss Baker, of Nightingale Lodge, St. Thomas's Hospital.—The annual meeting of the Boys' Surgical and Convalescent Home, Banstead, Surrey, was held on February 25th. There were 21 inmates at that time, and only one death had taken place during the year.

### Post Graduate Clinics for Nurses.

### THE USES OF WATER IN THE SICK-ROOM.

### BED ABLUTIONS, SPONGE AND SITZ BATHS.

THE treatment of the sick by spongings, packs, and baths is growing so strongly in favour that it is well for the nurse to be conversant with the newest and latest forms of bath and water treatment. Douches, lavages, and water applications of varied nature are so much used in the modern sick-room that it will doubtless be instructive to the hospital as well as the private nurse that the various valuable purposes which water serves in treatment should be set forth in these clinics. Water in the form of vapour baths and inhalations is commonly prescribed, and, altogether, the simple element furnishes so many useful ends in nursing that some practical hints as to its multiple applications will not come amiss to any nurse. Every nurse realises the value of soap and water, from the mere cleansing effect of the daily sponge bath up to the more complicated wet-packs, fever baths, cold sponging, and vapour treatment. Considering the constant and important part played by the "bed ablutions" of the sick, any little wrinkles and novelties dealing with these are an acquisition to a nurse's list of accomplishments. For even among the better classes the private nurse frequently has to combat the old-fashioned prejudice that while frequent bathing may be acknowledged desirable for the healthy, it is suspected that this is attended with mysterious danger to the sick. Now, as every nurse knows, the excretory action of a clean skin is even more important to the sick than to the strong, and the bath may assume a grave importance in saving life. And apart from its cleansing property, the use of water both internally and externally acts as a tonic, and is attended by a valuable change of body tissue. To give the daily sponge bath essential to the sick person in private practice, it is well to put a screen between the patient and the preparation of all the paraphernalia necessary to ablutions in bed. Most sick people dislike the fuss and trouble involved in the daily sponging, and it minimises the disagreeables if the weapons belonging to the bath are concealed from view. If the water is unpleasantly hard a water-softener may be with advantage employed, as, for instance, an allowance of one or two drachms of borax to a generous tub of water. This has also an antiseptic quality essential in some cases. Bath salts, eau de cologne, a few drops of spirit of lavender, and any other dainty addition to the bath may be made according to the custom and inclinations of the patient. A very small quantity of ammonia and cologne is a fragrant addition to the water after the face has been washed. No artificial agents, however, will suit the more delicate face skin. Toilet vinegar or common vinegar in the proportion of an ounce of this to each gallon of water proves very refreshing, and methylated spirit, where the patient does not object to its strong odour, serves not only as a refreshing and stimulating addition to the bath, but has an excellent property of warding off a possible after-bathing chill. The patient should lie between blankets, be provided with a pillow, and, if at all exhausted, some hot nourishment, preferably with a little stimulant, should be given 20 minutes or half an hour before the sponge bath. A hot-water bottle should always be put to the feet after the bath if the patient be elderly, exhausted, anæmic, with heart weakness, or in any other condition likely to be attended by chilly reactions. The fresh clothing, aired and warmed, should stand in readiness before the bathing begins, and a jug of very hot water be close at hand to keep up the temperature of the sponge bath. When the patient is not seriously ill or exhausted, it will be beneficial to allow the bath water to gradually cool down during the sponging process, but in serious cases the patient has not enough vitality to rally in reaction, and the temperature of the bath needs keeping up by the addition of hot water. The face should always be bathed first, then the chest, arms, legs, back, and the abdomen last, using a soft warm towel and some amount of friction, graduated to the condition of the patient. Friction, however, should not be employed over the abdomen unless specially ordered. It is well to give the bath quickly, but the patient should never feel that he is being hurried. A sponge bath should never be given until at least two hours has elapsed after a full meal, light nourishment not counting in this category. The tepid bed-bath may be given during menstruation, while cold sponging is not desirable at such a time, even if the patient while in health has been accustomed to it.

In the giving of all baths the nurse should exercise delicacy and prevent any undue exposure. In gynæcological cases the hip bath is constantly ordered. It is employed, too, in abdominal and visceral diseases, hæmorrhoids, constipation, and urinary disorders.

A practical point with regard to the sitz bath, whether hot or cold, is that the patient should wear a flannel nightingale or chest wrap, since the upper part of the body is apt to become very chilly, especially if the sitz bath be taken cold. Care should be taken that such a flannel protection is too short to reach the bath water. The nurse should in every case carefully ascertain from the doctor the temperature of the water and the duration of the bath, for a hot sitz bath may prove injuriously exciting if too prolonged, while if cool or cold it may, if the patient be kept in too long, become a powerful sedative when a tonic effect was intended. Another practical point for the nurse is that the room in which a patient takes a bath should invariably be warm, even though the bath taken be a cold or cool one! If the patient during the sitz bath does not wear a flanne wrapper on the upper part of the body, the nurse should put a warm bath towel over that part of the bath the patient's back rests on, otherwise a chill may very readily be taken. In some cases when the household possessed no sitz bath, I have used an ordinary full bath, the patient sitting in a low wicker arm chair placed in the bath. I have then gradually filled up the bath till the hips were entirely covered, and have arranged the feet resting on a towel slung across the bath after the fashion of a hammock. I can assure any nurses who follow this plan that it would be difficult to imagine a more delightful and comfortable method of taking a sitz bath. An ordinary cane-bottomed chair with the legs cut very short may be used when an arm chair is not available, but it is not nearly so charming as the easy chair with its arm-rests. After use in the bath a wicker chair needs to be well dried in the sun or in a kitchen. It has just occurred to me, and I present the idea, if it has never before been used, to any enterprising inventor who cares to make use of it, that a properly constructed chair for use in the bath would meet a want, since very many invalids complain of the fatigue of sitting upright in a bath, with the alternative of leaning against the chilly bath back. And the feet slung in the towel hammock so as to prevent them from getting wet proves much more comfortable than the ordinary disposition of the legs while taking a hip-bath. In describing the arm-chair bath I spoke of the patient sitting in the chair while the bath is filled. I need hardly remind the nurse that the filling of the bath under such circumstances must be very cautiously done and the patient must not be left a moment while the taps are running. The slung position of the feet prevents any possible danger of too-hot water reaching the patient.

### Echoes from the Outside World.

### AN OPEN LETTER TO A HOSPITAL NURSE.

I am afraid that I cannot help sadness being the prevailing note of the echoes which reach you this Easter-tide. The fearful disaster which overtook the "Stella" with its load of happy excursionists seems to have come home so much to us all because we have so often gone forth under almost the same conditions to spend a happy holiday, returned with renewed health in perfect safety, and yet thought absolutely nothing of the risks we have run. Then, quite suddenly, a catastrophe like this falls upon us, and we realise with almost a shock how thankless and ungrateful we have been hitherto, and our hearts melt with compassion and sorrow for the bereaved. But one point in reading over the accounts of the wreck strikes me forcibly, and that is how different was the behaviour of the women from what, with a few exceptions, it would probably have been some years ago. Then a woman in terror was frequently a woman in tears, overcome with faintness, and making impossible and unreasonable requests. But on that fateful Thursday, though many versions of the accident have been given, all agree that the women were as brave as the men, bidding good-bye to their husbands at the word of command, taking off their own cloaks to cover others worse off than themselves, singing clearly and strongly to cheer the rowers through the darkness, and sharing the toil in every way possible. Assuredly, the better training and the growing independence of the modern woman, which some deplore, has brought, as might be expected, a greater strengthening of character and more power of self-control.

I know by experience how suddenly the Channel fogs creep up, and how hopeless it is to attempt to see anything through the thick white wall which surrounds the ship in a few seconds. It is not many years ago that a party of us were yachting near Guernsey on a beautifully hot autumn day. Without the slightest warning we found everything as much shut out from our view as if someone had hung up white sheets around us. The fog-horn kept sounding almost incessantly, and our pace was so moderated that we scarcely believed ourselves to be moving. The captain reassured our fears by saying that we had passed the dreaded Casquets, and probably the pall would lift shortly, but almost as he spoke our lives were threatened. A voice was heard distinctly on our port side, though nothing could be seen, a dull thud-thud reached our ears, and a dim, dark shadow seemed reflected for an instant on the white fog. The captain shouted, our horn blew lustily, answered by another, and then a sailor standing close to me said "Thank God!" The Channel steamer had passed us within a couple of yards!

Is it not remarkable how general the practice of giving Easter eggs has become in England? I was purchasing two or three the other day, and the shopwoman told me that for one which was bought eight or ten years ago she now sold twenty. Some of her best customers come two or three weeks before Good Friday, so that they may insure a good choice, and buy as many as thirty, ranging in price from a few pence to a good many pounds. The simpler kind are merely made of sweetmeat or filled with bonbons, but some of the more expensive sorts are perfect dreams of beauty. Silk and satin covered eggs as large as a man's head, with costly lace bows and ribbons, containing valuable gifts or dainty nick-nacks quite as often as chocolates. But by far the most lovely specimens come from the florists' shops. These, as well as being filled inside with "candies," as the Americans would say, are smothered outside with the most exquisite flowers arranged with lace and ribbons, and sometimes held in place by jewelled brooches. There is also an attempt to give the lucky children of this age another delight by introducing the Easter morning egg hunt so well known in Germany. Eggs of all sorts—hen's, chocolate, sugar, paper, silk—are hidden all over the house, or in the garden, if fine, and then the little ones are set to search for them. You can imagine the glee. Perhaps another year some kind soul, when buying a supply, will remember the children in some of the hospitals. An Easter egg arriving quite unexpectedly would give something to think of and play with for the day at least. Any novelty is always such a boon to a child, is it not?

One of the latest crazes of the smart society lady is to play at Bohemianism. The details are most amusing. A certain clique who possess, according to their own belief, artistic talent, have banded themselves together for the advancement of their art. They have hired a studio, engaged a model, and meet at regular intervals to sketch. Neither master, nor mistress, supervises their work, which is not, I hear, by any means up to exhibition level, but attired in beautiful frocks they sit before their easels with a palette and brush in their hands and a cigarette continually between their lips. Discussions of an animated character go on, but not as one might suppose on the subject of light and shade, perspective and foreshortening. Man and marriage, dress and dancing, seem the more engrossing topics, and nicknames are generally used by the members. A couple of sisters are Punch and Judy, another member is Orchid, a third is named after an advertisement she is supposed to resemble. But the tea is the funniest part of the performance. In order to feel thoroughly Bohemian these ladies, whose tea tables at home are probably laden with every delicacy, eat thick slices of bread and jam, and to honour a special guest add penny buns to their fare!

The decision of the judge in a recent divorce suit in America is bound to be of interest to all women. A man married a wife and then discovered, after the ceremony, that the lady possessed both a glass eye and a false leg, of which facts he maintained he was in perfect ignorance at the time that he married her. How any man in possession of his eye-sight could fail to detect the difference between a real eye and a glass one, a live leg and a cork one, is a problem difficult to understand unless the courtship had been unduly hurried. Anyhow, the magistrate affirmed that if the wife had never been asked before marriage whether all her members were her own she could not be accused of deceit, therefore there were no grounds for a divorce. He added that it was perfectly legal for a woman to improve upon Nature if she wished, and the wearing of "false hair and other falsities peculiar to females" could not be constituted reasons for a dissolution of marriage. The "other falsities peculiar to females" was rather unkind. Do no American men ever wear wigs, I wonder?

Do you know the latest addition to a baby's wardrobe? Anyone who has much to do with these small people will have noticed that already a bride's trousseau is nothing to a baby's layette, if you include short-coating as well as long clothes. But it appears that a society baby has really not enough garments to make itself smart when brought down at afternoon tea for inspection by its mother's guests. So some ingenious individual has invented an infantile tea jacket. This Bébé Tea Coat—or ought it not to be called a coat-ee?—is worn over the white frock to give a finishing touch to the darling's toilette. It is made in muslin over pink or blue washing silk, trimmed with beautiful lace and insertion; of white China silk, with innumerable little tucks; or of fancy cream material with lace and chiffon collar. The prices vary from 21s. to 45s., and the sizes are such as to fit Miss Baby from six months to two years.

### The Princess Mary Village Homes, Addlestone, Surrey.

About twenty-eight years ago Mrs. Meredith (well known for her work amongst discharged prisoners), seeing the great need of something being done to rescue the children of criminals from drifting into the vicious ways of their parents, founded the Princess Mary Village Homes. Her object was to save these little ones from a life of crime and bring them up as respectable members of society, able to earn an honest living. The homes were started especially for this class of female children, and mention is made in the Trust Deeds that any child whose parent or parents have been convicted of crimes shall be admitted in preference to other destitute children.

The institution, occupied on an average by 170 girls, is arranged on the plan of a small village. Entering the gates one faces a long oval green dotted with flower beds. Round this, but separated from it by a broad gravel drive, are grouped the various buildings that comprise the homes. In front of each cottage, over the walls of which are trained a variety of pretty creepers, is a tiny plot of grass and a flower bed. The first we enter after passing the gates on the left is the office. Here all business is conducted and children are seen by their friends. Passing one of the smaller cottages we come to the chapel, which lies somewhat back from the drive. A peep inside shows us that its chief characteristic is extreme simplicity.

On a Sunday the children, with their bright faces and neat clothes make a pretty and interesting picture. On leaving the chapel we enter a cottage which may be taken as typical of the rest. The door is opened by a bright-faced respectable young woman whom the occupants of the cottage call "mother." On the right hand we go into a fairly large and well lighted room called the parlour; it is decorated with pictures, photographs, and all the nicknacks that make a room homelike. Plants flourish in the long window, and flowers stand on the tables. Beyond this pleasant apartment is a sort of lavatory where the children wash and keep their boots, clothes, etc. Returning to the entrance we go into the dining room on the left. It is much the same as the parlour, only the long table, benches, dresser, &c., show the use it is put to. Everything is beautifully clean, and the tables and boards are white as constant scrubbing can make them. From this one passes to the pantry and the little kitchen. At the back is a brick yard, coal-house, &c. Upstairs the dormitories are of a good size; they are very clean, but look rather bare with their white uncovered floors and rows of little beds. The walls are plastered and distempered with blue, and hanging on them here and there are large texts. The "mother" has a tiny room to herself; the windows are open, and the place is well ventilated. A cottage of this size contains sixteen girls, but although they cannot all accommodate so large a number, yet the arrangements are the same. On leaving this pleasant little home we pass three similar ones on our way down the grounds, the last being known as the "Baby" cottage. A peep inside shows that the babies' playroom is large, sunny, and bright, and the three tinies who at present occupy it are having a rare game. Neither the dress, nor the looks of the children, nor the appearance of the room with its nursery comforts and wealth of toys, suggests the idea of a charitable institution. That the babies are well cared for is quite evident. Sometimes when they are first brought to the homes they are in such a deplorable state of neglect and ill-health that it is a marvel they live at all, but good feeding and careful nursing work wonders.

The next building, lying back from the drive, the space in front of which is filled with wet clothes, tells its own tale. Here is the laundry, where the washing of the institution is done by the bigger girls under a superintendent. Some work half, some a whole day, according to their ages. Now we come to the largest building on the grounds. It is at the end of the lawn and faces the gates. The centre part is the school, and the wings are used, one as a training home for the girls about to go out to service, the other to accommodate some of the officers.

A glance inside the school will show us where the majority of the girls are at present. The room or, rather, hall, is very large, divided by sliding doors to separate the classes. The faces of the children are a study; some pretty, some plain, some ordinary, but all looking healthy and happy. The infant school is a separate room at the back of the building. It is lofty, bright, and well ventilated. As we pass out of the side entrance we catch a glimpse of a large kitchen garden that more than half supplies the homes with vegetables. We then come through a pretty flower garden into the drive again. Leaving two more cottages on our left we make for the Infirmary. It is a long, low building, standing back by itself with a large lawn to separate it from the drive.

Entering the front door we find ourselves in a tiled passage running right and left. Turning to the left we go straight to the door at the end. This opens into the little dispensary. It is nicely fitted up and well stocked with drugs, &c. Here the little out-patients come every morning before school or work begins to have their ailments attended to by the Sister.

Leaving the dispensary we turn back into the passage again. On the left we pass the sisters' room and the "mother's" room, then we look into a small ward of three beds. The room is bright and sunny and looks out into the fields at the back. The walls are painted buff and brown, and the floors are stained. The beds have scarlet and white covers, and a curtained window looks into the passage. The next apartment is the kitchen, a nice little place with a good-sized range and boiler to supply the baths. From this we enter the day-room, used as a ward till the new wing of the infirmary is finished. It contains four beds. Leading from this is the lavatory and bath-room. The new wing in course of erection joins the infirmary at the east end near the dispensary. It will contain two rooms for the sister, a ward for sick workers, a long corridor to be used as an out-patient waiting-room, and the usual offices, &c. When it is finished the room now occupied by the sister will be put to its original use as a ward of three beds.

At the back of the infirmary is the Jubilee playground, a large field with a few fine old trees in it. Here the children have swings, &c., and many a good game they enjoy in fine weather. In the corner of the field, railed off from intruders, is a little iron building known as the Irene Hospital. It is for isolating any infectious cases. As soon as a child is known to be suffering from an infectious disease she is removed from her cottage, which is put in quarantine for a certain time, and nursed at the Irene Hospital. Following this plan prevents the spread of infection, which would otherwise so easily take place amongst so many children. A glance at the inside of the isolation hospital shows a compact little iron building lined throughout with light-coloured varnished wood. The large ward contains four beds, but would accommodate twice the number if necessary. At one end is the patients' entrance, w.c., &c.; and at the other, (separated from the ward by a passage) is the nurses' room, warmed by a gas stove, a lavatory, kitchen, scullery, and pantry. The kitchen is fitted with a gas cooking stove. Before leaving the subject of the infirmary I should like to say a few words as to the children's health. On the whole it is excellent. The sister of the infirmary daily visits each cottage and inquires of the mother as to the health of her family. Every child, at the first ign of illness, is brought to the sister, who exercises her judgment as to admitting her to the infirmary, sending for the doctor, or waiting till his usual visiting day. In every way, physically and morally, the children are watched over and cared for and made more equal to fighting the battle of life when they are obliged to leave the shelter of the homes.

The next place to be inspected is the sewing-room, presented by the Duchess of York, who has graciously consented to be patroness of the institution in place of her lamented mother. Here all the clothes of the children are made under the direction of a qualified superintendent. The girls are taught to cut out and make their own clothes. Orders for fine underclothing are gladly executed. Many a dainty little undergarment has been made for the royal children of their patroness by the hands of these poor girls. Turning from the sewing-room we pass two more cottages, the meat house, coalyard, &c., and enter the stores. Here we find a place like a large general shop, presided over by a lady assisted by two girls. Everything is here, meat, vegetables, grocery, bread, and other things too numerous to mention. At stated hours a bell rings and mothers and girls go and fetch the provisions for the day or week as the case may be. We notice an excellent apparatus for Pasteurising the milk before it is served out to the cottages. By the way, there is a strict rule that only boiled water is used for drinking purposes in the homes. The diet of the children is plain but good, and they have plenty of it.

Over the provision store is another larger place where all the clothing is kept. There is now nothing more to be seen, for on leaving the stores we find ourselves at the entrance gates.

Addlestone is only twenty miles from London, and the homes are well worth seeing. Visitors are welcome any afternoon from two p.m. till five p.m. except on Saturdays.

### Free Libraries and Anti-Vaccination.

BY LADY WATKIN WILLIAMS.

"CONSCIENTIOUS objectors" to vaccination are becoming so numerous as to alarm all intelligent persons who can appreciate the cruel edge of the sword they threaten to let fall on coming generations. The people of England—as a mass—seem now, as ever, to justify the old hard sentence on them, "mostly fools;" for they gaily follow their leader down an all unknown, because all unrealised, road, incredulous that at the bottom lies death in the most revolting, and once the most dreaded, guise that our last grim enemy can assume. Scientific men occasionally say an ineffectual word, but mostly look on in sad silence, having found by experience that their weapons of reason and demonstration fall down blunted before the ignorance and unscrupulous audacity which in words appear to win the victory.

And yet, though silenced, they are still eager to persuade the people of their folly, to save their helpless children, if not themselves, from the cruel curse they so rashly call down upon them. But the pity of it is that the unequally matched forces never really come in contact with each other; the conviction of the one never gets near the heart of the other; and partly from the Englishman's justly rooted dread of any interference with the liberty of the subject, partly from his ingrained antagonism to the claims of any esoteric wisdom, and largely from his keener appreciation of occasional consequent personal ills than of general consequent doom and suffering, the man of the people holds on his misguided way, defiant alike of argument, of warning, and of threat.

The lead once given, love of notoriety lends its evil aid, and the few scattered followers become the crowd of active supporters. What proud young father would knowingly expose his lovely laughing babe to the fearful destroyer? And what intelligent father would fail to become conscious of the risk if proofs were only brought home to his understanding? But loud talk wins its ignorant votaries, and the pity of the anti-vaccination movement lies chiefly in the fact of the blind ignorance of its supporters—an ignorance often frankly confessed in the course of friendly discussion. If, indeed, the objectors were all truly and deliberately actuated by "conscience" one would still protest against the reckless sacrifice of those who have no power to protect themselves, but could not refuse respect to the objector. In this case, however, it is clear that the majority of objectors have been guided neither by conscience nor reason, but have only adopted the catchword of their leaders, and are acting not from conviction but from the ever-contagious spirit of assertive independence.

Can nothing, then, be done to reach these mistaken men of impulse? and is there no help to be found through the medium of the free libraries? The report of the Royal Commission of Enquiry, issued in 1896, comprises five most important appendices, which contain detailed accounts of certain small-pox outbreaks in London, Warrington, Dewsbury, Leicester, and Gloucester, with analytical tables and diagrams which speak for themselves at a glance. They were compiled by experienced physicians appointed for this purpose by the Government, who spared no pains to render their reports full and exhaustive.

These reports can be obtained either in combination or separately at moderate cost; but the suggestion is here made that the libraries should claim to have such Blue Books presented to them officially *pro bono publico*.

The reports may at first sight appear to offer too voluminous a mass of observations and deductions for the digestion of the popular reader; but many of the yet blind followers of this fatal prejudice are not mere popular readers, and would not, in face of so serious a subject, refuse to study the whole evidence when set before them.

If these reports (and it seems obvious that they are the just due of the people for whose benefit they were compiled) could be laid on the reading-room table of every free library in the land, it is inconceivable that an impression would not be made beyond the power of all words to create, and that large numbers of our fellow-men would not be brought to the touching cry of hospital sufferers in Gloucester, "Oh, warn our dear ones at once to accept the vaccination that we refused."

Surely this would be a lasting and valuable use to make of the libraries; a use which would commend them to their detractors. It would also rejoice the hearts of those who welcome any outlet for thought and interest to their fellow-creatures, and who warmly support free libraries from the conviction that they alreadv, to a large extent, fulfil this object.

### Where to Go.

THE DOWDESWELL GALLERY, 160, NEW BOND STREET.—An interesting exhibition of the original drawings from the Rubaiyat of Omar Khayyam, and other drawings, sketches, and oil-colour paintings by Elihu Vedder, is now on view at the above galleries.

### Our Convalescent Fund.

THE way in which our readers are rallying round our—or, rather, their—Convalescent Fund is very cheering. This week we acknowledge with thanks 3s. from Nurse Matthews; Nurse L.B.R., 5s.; Nurse E. Lucas, 5s.; Policy No. 1,081, 2s. 6d.; and 5,072, 2s. 6d. The last contribution is particularly pleasant to receive—it is a gift in recognition of the help given to a convalescent nurse

### The Ethics of Nursing.

GOLDEN SILENCE.

WE nurses have in these days ample opportunities of acquiring knowledge of our profession. Our hospitals, great and small, lay themselves out to train their nursing staffs with thoroughness in every department of their work. But it has dawned upon me of late that in many cases a training in what I call, for want of a better designation, the ethics of nursing, has been neglected. We have been taught how to wash a patient and make his bed; we are drilled into habits of punctuality and accuracy and tidiness. We can dress wounds and care for backs—and, in fact, do all the practical, that comes to us every day, well and thoroughly.

But the longer I live and the more I see of nurses from an outsider's point of view—especially in private nursing—the more am I convinced that something is still wanting to bring training to an even greater point of perfection. We do not require our nurses to be merely well oiled machines! We want extra superfine human beings.

It so happens that I have lately come in contact with a nurse who is partially the text for my remarks of to-day. She was well trained in her work—she was devoted and unselfish; but she left much to be desired in matters about which, perhaps, her hospital sisters had not instructed her.

She had not learnt the virtue of golden silence! She did not remember, or at any rate she did not heed, the great maxim of the wise man, "There is a time to keep silence, and a time to speak." "A time to keep silence"—if only nurses would study the importance of that motto.

With the nurse of whom I write talking was absolutely a disease, and, like the stream of which the poet sings, she "went on for ever." But, oh, quaint delusion, she thought that she kept her patient quiet, and only spoke when necessary. She prattled ceaselessly, whatever she was doing, and whether it was night or day; but when the medical man impressed upon her the necessity for extreme quiet she merely said, "Oh, yes, I never talk when I take my patient anything; I just give it her without a word." This was, as I say, a blissful delusion.

The patient was desperately ill, the babbling flow of conversation to which she was subjected nearly maddened her. Ceaseless, aimless chatter is sufficiently annoying when you are well—when you are ill it is like pandemonium. This good nurse talked of her other private cases, and of her own wonderful way of nursing them. She prattled of hospital, and had a pleasing habit of repeating rather nauseous professional tales when the patient was trying with difficulty to swallow food she neither wished for nor liked, under any circumstances, at the moment. She had an unpardonable custom of asking the advice of the patient as to that patient's own treatment. Now although the said patient had been a nurse herself she was only anxious now to be treated as a patient pure and simple, and did not wish to be asked to suggest cures for sickness, nor to have to select her own diet or to prescribe what was to be given her if she felt faint. Even in the dead of night, that talkative nurse rarely brought her even a small quantity of milk or beef tea without accompanying yards of explanation as to the why and wherefore of the meal. To be awakened widely and entirely to listen to quite unnecessary explanations about food you would rather not have, and if you must have would fain swallow down quickly and quietly, is, to say the least of it, trying to the temper.

Ah! silence is very golden in a nurse—especially at night. Why in the world cannot the food be brought silently to the bedside and without one unnecessary word? As long as your patient takes it, you do not want to wake them right up in the process.

It is surely perfectly easy to say a bright word or two when necessary, but few things are more irritating to a sick person than the constant strain and tension of feeling, "Now she is going to talk." A perpetual stream of chatter keeps up an unrestful atmosphere about your patient; it gets upon his nerves, and the strain and worry may be, and often are, most serious drawbacks. There is something soothing and restful about a silent, gentle person, who talks if you are inclined to talk, but who is otherwise quiet; and I should like to impress the value of silence upon all those nurses who are born chatterboxes. That little unruly member of ours can be ruled even by the most talkative among us.

"Oh, if nurse would only be quiet, if she would only hold her tongue; I cannot stand that clacking voice," I have heard a patient say. "Speech is silver," yes; but "Silence is golden." Can we not cultivate, as we cultivate the other qualities that go to make up a perfect nurse, this by no means unimportant virtue of "golden silence"?

### Presentations.

MISS AGNES MACDONALD, late sister-in-charge of the wards of the Royal Aberdeen Hospital for Sick Children, has, on the occasion of her leaving, received many gratifying tokens of esteem and goodwill from those with whom she has worked for the past six and a half years. The medical and surgical staff presented her with a beautiful solid silver tea service, the nurses gave two very handsome silver corner dishes, also silver butter knives, while from other friends she received various useful and pretty gifts. Miss Macdonald carries away with her the hearty good wishes of all her friends in Aberdeen.

MISS PIDGEON, on her resignation of her post as matron of the Jaffray Hospital, Birmingham—which she has held for nearly eight years—was the recipient of some valuable gifts, including a handsome gold and platinum brooch from the nursing staff, and an afternoon tea kettle and stand from the servants. We understand that Miss Pidgeon is about to take up work in Edinburgh, where she will carry with her the good wishes of a large circle of friends and of all those officially connected with the hospital, coupled with many expressions of regret at her departure.

LAST week a testimonial was presented to Miss Kerr, the assistant matron of Lambeth Infirmary, who has resigned her position. The matron (Miss Griffiths) presided on the occasion, and there was a numerous attendance of nurses. The chaplain (the Rev. T. H. Leale) having been called upon by Miss Griffiths to give expression to the thoughts and feelings of the subscribers briefly addressed the meeting. He referred to the fact that though Miss Kerr's official career in the infirmary had extended to but little beyond three years, yet the influence of her high character and pleasing disposition had made a deep impression during that short period. He also spoke of her popularity among the nurses and patients, and pointed to the long list of subscribers and the remarkable liberality of their contributions as an evidence of the fact. Mr. Leale then pleasantly touched upon the circumstance that Miss Kerr was not leaving for the purpose of taking a similar position, but said she was about to be married, so that the intended gifts must be regarded in the light of wedding presents. On behalf of the meeting he wished her all happiness and prosperity. The presentations consisted of a solid silver tea service of elegant design, the gift of the medical officers and of the nurses; a solid silver salver, the special gift of the matron; a pair of silver jam spoons from Dr. and Mrs. Quarry; and a fine cut glass water jug, the gift of the servants of the infirmary. Miss Kerr, in well-chosen and appropriate terms, thanked the donors for their kind wishes and valuable gifts.

### Across the Seas.

### SOUTH AFRICAN HOSPITALS.

### I.—"Nigger Work."

The entire system of hospital management in South Africa is passing through an interesting and somewhat experimental transition period. We are young beginners at hospital administration, and like all beginners we oftentimes find it necessary to retrace our steps and take down brick by brick a laborious system which has been set up, but which has proved not to fit in with colonial custom.

Inquiries are made so constantly by English nurses as to the field offered to their energies by the virgin hospital ground of South Africa that some particulars as to the nursing life and its variations may prove of interest to such inquirers.

From the letters received by the hospital matrons of this colony from English nurses it would appear that the latter are under the impression that South Africa is a land flowing with milk and honey, a kind of Klondyke in hospitals, where every kind of joy and felicity awaits the nurse, trained or untrained. The simplicity of some of these letters cannot fail to rouse a strain of sincere sympathy in those who receive them. But the totally erroneous and gilded views of the majority as to the position of the hospital nurse in South Africa renders it desirable that some definite and practical information on this subject should be given to English nurses. To those who think of leaving the known path of hospital ways to try their fortunes in a new colony, some definite information as to South African customs may be of importance. To home-staying hospital workers the conditions of work and life abroad and the struggles and aspirations of their colonial sisters in attempting to raise and evolve the nursing profession should prove equally interesting, as strengthening the chain of sympathy which binds nurses together throughout the civilised world.

My readers must please bear in mind whilst reading my criticisms and comments that I am putting the case entirely from the colonial point of view, and, as we shall see presently, this does not always accord with established English hospital traditions.

The first word of advice one might offer to an English nurse who seeks her fortunes in South African hospitals, as her brother seeks it in the mines of Kimberley or in far-off Rhodesia, is that much of her success will depend on how far she has been able to free her mind from insular prejudices and the cut-and-dried convictions of an older civilisation than ours. And it is just in this particular point so many nurses fail. Britishers in general, as we learn from history, seem to find that fundamental principle "other countries, other customs," most difficult to grasp. And we in South Africa have suffered considerably from this limitation.

English methods of hospital management, pure and simple, without the modifications which racial and climatic conditions render necessary, have been imported and planted right down in our midst, and have proved a distinct stumbling-block to progress in the development of our South African hospitals. You cannot graft English methods, unaltered and undiluted, on to the colonial system. The rigid rules and regulations which work most admirably in an old-established London hospital, in an old-established country, fall to pieces, and prove in the working to be utterly unsuitable to a pioneer, unfinished, and developing colonial town which has yet to make its history, its standard and its guiding rules.

The hours, the regulations, and the entire programme of a London training school have been repeatedly introduced into the hospitals of out-of-the-way mining towns and newly sprung-up communities here, and the result has been failure. To transplant to an out-lying African town the finished and perfected curriculum of a leading English hospital is to court failure and to entail a great deal of unhappiness on a colonial nursing staff.

There is no question that individual instances serve better than any amount of theorising to point the moral of an argument. I will therefore particularise a few examples of the direction in which trouble and friction have arisen from an attempt, all at once, to force the young colonial idea to shoot, instantaneously as it were, toward the English ideal. Mind, I have nothing to say against the English ideal of nursing. Indeed, all South African nurses who are ambitious of raising their profession and improving colonial methods of hospital management have nothing but admiration for all that has been accomplished in the nursing world of England. But all of us who think, readily appreciate, as a practical fact, that English ways need to be modified and watered down to suit the social conditions of colonial life. There are so many and such striking differences between the modes of life here and those obtaining in England that it is impossible to make the one system fit the two countries.

English hospital systems suit England because they have slowly and gradually grown up in the country. And the fact that they originated in England proves that there was a necessity for them. In the hospital system, as in national customs, the law of natural selection is in operation, and this leads to the choice of those things which are suitable, and the rejection of those which are unsuited to the special needs and requirements of particular countries.

But just as many English men in tropical climates insist on wearing the silk hat and frock coat of Piccadilly, so many English nurses and matrons coming out here have insisted, against common sense and good advice, in forcing on the colonial hospital worker a *régime* which does not fit in with colonial environment. To come to practical facts, I am bound to record that trained nurses throughout South Africa are convinced that an immense amount of harm has been done to the nursing profession here, and that progression has been materially held back by the introduction into South African hospitals of that English custom which relegates a considerable percentage of the sweeping, cleaning, and scrubbing duties to a nursing staff.

In all countries where coloured races exist the performance by white people of "nigger work" proves to be a mistake At any rate this is so in South Africa. The Kaffirs cease to feel the respect towards white nurses which it is prudent should be inculcated in the race generally, when they see the hospital staff performing duties which a "superior nigger" would flatly refuse to perform.

The scrubbing and cleaning which apparently has lent itself to the curriculum of English training schools, and has improved the race of nurses, has certainly been a terrible mistake here. The performance of such work has resulted in nurses "losing caste" in native eyes, and this circumstance makes it hard for a white nurse to keep proper discipline in her wards.

Before the advent of the English-trained matron and nurse household work was performed, as it always should be, and is in all tropical and sub-tropical countries, by the natives. The fact that a large share of this domestic work now falls to the share of the nurse in Anglicised South African hospitals has occasioned a disastrous difference in the social status of trained nurses. This one circumstance that hospital nurses are expected to perform "nigger labour" has kept the best colonial women from training as nurses. For the woman who has been imbued from childhood with the view that scrubbing and cleaning is Kaffir work cannot easily and readily divest herself of this early and natural impression. She hesitates, although she may have a keen desire and a natural aptitude for nursing, to place herself on a level, during her training, with a Kaffir sweeper.

### A Crusade of Cleanliness.

WITH hospital, district, and private nursing we are all familiar, and now we must accustom ourselves to the thought of the nurse in the school. There are in London alone 850,000 children attending the elementary schools, and the vast mass of these children are (to put it plainly) unhealthy and unclean. They come from crowded districts and homes where cleanliness is practically impossible, and so we get the report of the Duke of Norfolk that nearly every girl who presents herself for Post Office work is found to be suffering from a dirty head. This state of things is insufferable, but teachers and district visitors have tried in vain to cope with this question of vermin, and at last it has been recognised that a trained nurse to visit schools in poor neighbourhoods is an absolute necessity. But the question of vermin, though of the utmost importance, is only one of many questions; next to it stands the prevalence of contagious eye disease (also caused by dirt), frequently leading to blindness; and then we have the whole mass of infectious illnesses from whooping cough to diphtheria, which are undoubtedly spread by the congregation of children in class rooms. All contagious and infectious illnesses are a disgrace to civilisation and a shame to science. The condition of the homes of the people in large towns, the prevalence of vermin which infest these homes, and the lack of teaching on homely subjects is a reproach to our housing and educational authorities. If there is one thing that the public health demands more than another it is that our schools should be supplied with baths and with all means of attaining and teaching cleanliness to youthful citizens who may perhaps be trained to be superior to their parents. Now the district nurse has no time to visit for such ills as these—ills which affect not only whole streets and blocks of buildings, but whole districts—and the only way to tackle the difficulty effectually is to treat the children in the schools. In 37 of our London schools—37 out of about 600—a nurse visits regularly twice or three times a week. One of these nurses, working in Chelsea, says that she finds in about six months she stamps out all the cases of eye disease and dirty heads in a school. This is the most remarkable evidence of the value of a nurse's work that it is possible to have. Compare with it the statement of the head teachers of many of the girls' schools, who say that about 90 per cent. of the girls in poor neighbourhoods have unclean heads. Of course an occasional case of eye disease or vermin often re-occurs in a visited school, but it is attended to at once, and so long as the nurse visits regularly the old state of dirt cannot exist.

On the value of the work done in regard to arresting the spread of measles, scarlet fever, mumps, and diphtheria, no statistics are available. But a bad outbreak of any of these diseases has never occurred in a visited school, and the nurses bear witness that they have all had children with mumps, diphtheria, measles, &c., brought before them, and have, of course, promptly sent the children home; in these cases there has been no epidemic running through the school.

Again, the nurse is able to treat the innumerable small sores from which unhappy children suffer; if she is wise she gets the doctor under whom she works to allow her to use mercuric ointment, for, whatever the sores may arise from, the taint of congenital disease is generally to be suspected.

This, of course, makes it necessary that only the most highly-trained nurses should be employed for school work, for it is only to the most highly-trained and therefore most humble of women that this powerful poison and specific can be entrusted. Where corrosive sublimate is not allowed as a disinfectant and ointment, boracic or carbolic are used instead. The school nurses in London are at present working mostly from charity, but it has been felt by those who are interested in the health and progress of London's scholars that it is time this movement should be put on a businesslike and thorough basis. A London school nurses' society has therefore been formed, of which the vice-chairman of the School Board is chairman of the committee, and which has the following vice-presidents: The Countess Grosvenor, the Countess of Dartmouth, Lady Beatrice Pretyman, Lady Windsor, Lord Reay, and Sir Henry Burdett. The committee consists of Mrs. Leon, who first started a school nurse in Bloomsbury; Miss Rosalind Paget, Mr. Brudenell Carter, Dr. G. E. Shuttleworth, and the following members of the School Board for London: Earl Beauchamp, Mr. Bridgeman, Mrs. Homan, Mr. Scott Lidgett, and Miss H. Morten. The committee have decreed that where possible they shall simply raise the funds and pay for a Queen's nurse from the local centre to do the work, but the East London District Nursing Association and the Haggerston and Hoxton Association have declined to lend their aid, so in these two districts the society has started its own nurses. Meanwhile in Bloomsbury, fostered by Miss Gray, in Hammersmith, fostered by Miss Curtis, and in Southwark, fostered by Miss Shallard, the work goes steadily onwards. Other centres are eager to supply nurses to do this great preventive and cleanly work, but the society at present lacks funds to pay for all the nurses needed. And yet the sum is a small one; probably sixteen nurses at a cost of £800 a year would meet this terrible evil, for it must be remembered that when once a school has been regularly got into order the nurse finds her patients dwindling down to the disappearing point; and as a school nurse can only work during school hours—that is, five hours on five days a week during term—her whole time is not needed, and £50 is decided to be adequate salary. Adequate reward from the people of London it would be impossible to estimate. Prevention is better than cure, and how much a school nurse saves the ratepayers cannot yet be said; but we all know that children fill the fever hospitals, and we all know how costly these hospitals are. It seems only reasonable to conclude that £800 spent on school nurses would save £8,000 spent on hospitals. The address of the treasurer of the London School Nurses' Society is W. C. Bridgeman, Esq., 89, Harley Street, London, W.

### Resignations.

IN consequence of her marriage Miss Curtis, Matron of the Hospital Convalescent Home, Park Wood, Swanley, has resigned her appointment. She has filled the office with great credit to herself and to the entire satisfaction of the trustees since July, 1894, and her loss is much regretted by all.

WE learn that Miss Isabel Firth has resigned her appointment as Matron of the Cottage Hospital, Gorleston, Great Yarmouth, which she has held for the past three years. She intends to retire into private life, and will in future make her home with her sisters at Wakefield. Miss Firth has had nineteen years' experience of hospital work, and has been a subscriber to THE HOSPITAL since its first issue. We beg to offer her our best wishes for long life, health, and happiness in her well-earned leisure.

### Minor Appointments.

BOROUGH OF DOUGLAS ISOLATION HOSPITAL, ISLE OF MAN.—Miss Mary Smith was appointed Nurse for sole charge of the above on March 22nd. She was trained at the Parish Infirmary, Brownlow Hill, Liverpool, and she has since been charge nurse of the City Hospital North, Liverpool; charge nurse of the Eastern Hospital, Metropolitan Asylum District; and matron of Eston Sanatorium, Eston, *via* Middlesborough.

### A Book and its Story.

"ANGEL: A CORNISH ROMANCE."

Mrs. Ensell's recent romance* is one that must appeal to all who appreciate vivid descriptions of wild coast scenery, mingled with scraps of folk lore and the weird romance, inseparably associated with stories of the old smuggling days on the Cornish coast. The sound of the sea is in our ears, and the freshness of the brine blows towards us with an invigorating, stimulating touch, as we turn the pages of her book.

The scene of the opening chapter is laid in the waning hours of the last century. A wedding party is gathered within the walls of a village church in a remote country parish in the North of England. "The Vicar's beautiful and only daughter was the destined bride, and the bridegroom the squire of the parish, a gentleman of large fortune, who had recently come into possession of his property on the death of the late owner. Whether the bride was destined to be happy or not, certainly the sun did not shine upon her; a leaden sky and a drizzling rain darkened the atmosphere and depressed the spirits."

Wedding parties are not proverbially cheerful assemblies, and the gloom of this particular one deepens into anxiety as the slow moments of waiting pass and no bridegroom appears. "The young bride in her thin white garments looked pale and scared. The Vicar passes in and out, the young people whispered and tittered, the old ones shook their heads and grumbled." Facetious remarks are passed as to the possible cause of the bridegroom's non-appearance; but, in spite of these usual devices to beguile the tedium of waiting, he fails to appear.

In the meantime the conscious cause of all this perturbation is pursuing his way south with all the haste which the slow modes of travelling permitted. To sever himself at once and for ever from the scene which until a few hours previous had held all that made life dear was the determination arrived at after a night of restless uncertainty. That he had his own "reasoned reasons" for thus acting the reader will find when pursuing the story for himself. He arrives, "after two days of exhausted patience and aching bones," at a cottage in Cornwall, overlooking an isolated rock-bound Porth or bay. He is received by its owner with the suspicion natural to natives of a district where strangers were rare. But Howard's appearance disarmed suspicion; he looked honest, he was not a "painter ' (a being above all others to be avoided by the cleanly, thrifty housewife), and so, after a slight exchange of amenities, he settles down in his new quarters. His attention is called to an inscription on the window-pane, "John Wesley, June 17," cut, or rather scratched, on the glass.

"Ah! I see—a sacred relic."

"Yes, you may well call it that, my son, and thankful may you be for a sleeping place under the roof that once sheltered the saint of God."

Amid the sublime scenery by which he is surrounded, he finds, as so many wounded hearts have done before, alleviation and distraction for his sorrow. For here was "solitude without loneliness; a climate that braces while it soothes; a grandeur that is rarely oppressive; endless variety, and a life and spirit in the outer form of nature that penetrates the being and stirs into new vigour the dormant faculties of the most sluggish and love-begone mind."

In this remote and thinly populated district he was surely safe from encounters with cultivated beings. But one day in his wanderings around the rock-bound coast he catches a fleeting vision of loveliness, followed later by "the sound of a woman's voice, rich and sweet as that we hear in dreams, or dream of in our waking hours under a poet's spell. An echo took up the tone and seemed to carry them to a region far remote from mortal men, and then rich and full they broke again through space and filled the yielding air." This is decidedly disconcerting. What man could be proof against such alluring sounds—certainly not our hero, described as "a man of many emotions." So incontinently following the sounds he traces them to the entrance of a cave, listening spellbound to the closing notes of the song, and waiting and wondering to whom the lovely voice belongs, he finds himself confronted with the fair "Angel" herself, in company with her beautiful mother. Both were foreign-looking, with a touch of Spanish blood, as Howard saw at a glance. Their appearance is explained by the fact that the place of encounter is really the entrance to the subterranean castle in which the outlawed owner of the adjoining Penwarne estate has his present abode. An acquaintance springs up between Howard and the occupants of this eerie stronghold, and he finds unusual interest in the story which Mrs. Penwarne unfolds to him of her past romantic and adventurous life, a period with which he finds he is closely connected, and her present one of servitude and imprisonment with her outlawed husband. Howard is called into the presence of the Penwarne, who desires his signature to his will. Here is a picture of the smuggler chief dragging out the remnant of his days in slow agony.

"He was propped up upon his couch with a cane-work bed-rest at his back, and a garment that might have been royal, for it was crimson silk bordered with ermine, thrown carelessly over his nightgown. His face had doubtless been handsome in its youth, for the line of his feature was fine and his eyes gleamed from under his heavy brows with the fire of a vigorous and undaunted spirit, but a sabre cut disfigured his forehead, and a similar gash across his nose and upper lip deformed the lower part of his face and gave him a savage and repellant aspect." The will is signed, and Howard's attention is called to a long marble chest concealed in an adjacent recess in the rock, hidden by a costly silk fabric in green and gold, which on being drawn aside revealed the chest.

"You would never guess what that is, now. It is my coffin. Where do you think it was made? In Venice. You can see it is foreign by the shape, and my name is on the top. But no date yet. It's not far off though. No musty mouldy earthen grave for George Penwarne, the outlaw and smuggler. What business has he amongst dead fellow creatures who cast him out whilst he was alive? No, no! The seamews will sing my requiem, and the wild blue ocean that I love will shed its salt tears upon my tomb." In the gloom of the scene moves the fair Angel, a ray of light in this dark chamber, and her sweet voice sings at Penwarne's request a little wild pathetic song suited to the environment.

The book closes with a vivid description of a storm and the wreck of a ship lured to its fate by a phantom light made by attaching a lantern to the neck of a horse and tying it to its fore leg—a common device used by wreckers for the destruction of many a gallant vessel. In that wreck the body of Sebastian, a man who throughout the storm has been the means of bringing acute suffering to more than one person, is washed ashore, and "Penwarne's subterranean castle was wrecked almost at the same moment as his ship, and his marble coffin mingled with other fragments of flotsam and jetsam that strewed the wild waters and the desolate shore." Upon the sale of his estate the proceeds were paid into the Exchequer, and the remainder of his ill-gotten gains went to public charities. Angel becomes the happy wife of Howard, and the gracious and beautiful chatelaine of his northern home. We cordially recommend the book to our readers.

* "Angel: A Cornish Romance." By Mrs. Ensell. (London: Digby, Long, & Co. 5s.)

### Everybody's Opinion.

[Correspondence on all subjects is invited, but we cannot in any way be responsible for the opinions expressed by our correspondents. No communication can be entertained if the name and address of the correspondent is not given, as a guarantee of good faith but not necessarily for publication, or unless one side of the paper only is written on.]

### THE STAFF-NURSE AND HER PROBATIONERS.

"CONSCIENTIOUS" writes: I am afraid that "Experientia" has made a somewhat sweeping condemnation of staff-nurses' treatment of their probationers, but I must admit there are some who quite forget their own days of probation, and that at one time they were as ignorant of hospital work and rules as their present probationers. I well remember my own days of probation, how leaving home for the first time, nervous and totally ignorant of everything concerning nursing, I entered a large county hospital, and was the next day placed in a male ward, which only increased my nervousness and made me feel absolutely stupid. The staff-nurse treated me as if I were quite beneath her notice, and rarely condescended to speak except to give a sharp reproof, though it would have been more to her credit if she had informed me beforehand what I ought to have done. A kindly word of advice and instruction would have set me at ease, but I could not endure reproof for doing, and leaving undone, things from absolute ignorance, so I left. My next experience was just the reverse. Not only staff-nurses but the matron encouraged and instructed me, telling me kindly of my mistakes, so that in time I became what the matron described as a conscientious, good nurse, and a capable woman. There always will be some probationers who are careless and thoughtless and try the patience of staff-nurses, but if they would only remember their own days of probation they could encourage and make happy even the careless and thoughtless pro's. There will always be a certain amount of favouritism; it is the same in public and private schools, in nursing institutes, and in our own homes there is often a favourite son or daughter.

### Appointments.

LANCASTER CORPORATION SANATORIUM.—Miss Annie Findlay was appointed Matron of this institution on March 9th. She was trained for three years at Brownlow Hill Training School, Liverpool, and has since been theatre nurse at the Stanley Hospital, Liverpool; district nurse at Stone, Staffordshire; charge nurse at Mill Road Infirmary, Liverpool; superintendent nurse at the Keighley Union Infirmary; and for the last two and a half years matron of the Keighley and Bingley Joint Hospitals, Keighley.

HOSPITAL CONVALESCENT HOME, PARKWOOD, SWANLEY, KENT.—Miss Campbell, assistant matron at the above Home, has succeeded Miss Curtis as Matron. She was trained at Newark Hospital for a year and a half, and entered St. Bartholomew's in 1892, where she obtained her certificate. After filling several temporary posts she was appointed assistant matron to the above, which she has retained until her present promotion.

CHEDDLETON ASYLUM, LEEK.—Miss Ada Arrowsmith was appointed Matron here on March 25th. She was trained for three years at the Liverpool Royal Infirmary, and afterwards she became successively staff nurse and district worker, and assistant matron at the Lancashire County Asylum, Rainhill.

TAUNTON AND SOMERSET HOSPITAL AND NURSING INSTITUTE, TAUNTON.—On March 22nd Miss L. P. Lessey, who was trained at the London Hospital, was appointed Lady Superintendent of the above. Her previous appointments have been charge nurse and matron of the Boston Hospital, Lincolnshire.

NORTHERN WORKHOUSE NURSING ASSOCIATION, 66, BARTON ARCADE, MANCHESTER.—Miss M. W. Kett has been appointed Secretary, in the place of Miss Lowndes, resigned.

### For Reading to the Sick.

AWAKE thou that sleepest, and arise from the dead, and Christ shall give thee light.—*Ephes.* v. 14.

**Verses.**

There lives
No faculty within us which the Soul
Can spare; and humblest earthly weal demands
For dignity not placed beyond her reach
Zealous co-operation of all means,
Given or acquired, to raise us from the mine
And liberate our hearts from low pursuits.
By gross utilities enslaved, we need
More of ennobling impulse from the Past;
If to the Future aught of good must come,
Sounder—and therefore holier—than the ends
Which in the giddiness of self-applause
We covet as supreme. —*Wordsworth.*

My life is in my hand, and lo!
I grasp and bend it as a bow;
And shoot forth from its trembling string
An arrow that shall be perchance
Like the arrow of the Israelite King
Shot from the window towards the east,
That of the Lord's deliverance. —*Longfel ow.*

Thou dost well in rejecting mere comforts that spring
From the mere mortal life held in common by man and by brute—
In our flesh grows the branch of this life, in our soul it bears fruit. . . .
Leave the flesh to the fate it was fit for! The spirit be thine!
By the spirit when age shall o'ercome thee, thou still shalt enjoy,
More, indeed, than at first when unconscious, the life of a boy.
Crush that life, and behold it's wine running!
Each deed thou hast done
Dies, revives, goes to work in the world. —*Browning.*

**Reading.**

If there be a sense of sin and want of spiritual blessings and a willingness to be saved by grace, though you know not that it is Christ's secret power that makes you willing, yet being athirst and willing you are invited. Do not puzzle and perplex yourself with such questions as these: Am I elected? Have I a right? Am I prepared? but come upon the invitation and take pardon, peace, righteousness, and every gospel blessing as free gifts to the needy. If one ready to perish with hunger and thirst were invited to a feast and assured of a welcome and he should hesitate, inquiring, Have I a right? Am I worthy? would it not seem preposterous in him thus to demur, when his necessities were pressing upon him and a plentiful table before him? The weary, the hungry, the thirsty, the guilty, the worthless, the vilest are invited to believe on Jesus, who came only to save sinners, and has assured them, in His Word, that they who thus come to him shall in no wise be cast out.—*Bogatsky.*

Thus saith the Lord. In their affliction they shall rise early to me; come and let us return to the Lord, for He hath taken us and He will heal us: He will strive and He will cure us. He will quicken us after two days; on the third day He will raise us up and we shall live in His sight. We shall know and we shall follow on, that we may know the Lord. His going forth is prepared as the morning light, and He will come to us as the early and the latter rain to the earth. "What shall I do to thee, O Ephraim? What shall I do to thee O Juda?" Your mercy is as a morning cloud and as the dew that goeth away in the morning. For this reason have I hewed them by the Prophets; I have slain them by the word of My mouth, and My judgments shall go forth as the light. For I desire mercy and not sacrifice: and the knowledge of God more than holocausts.

### Travel Notes.

By Our Travelling Correspondent.

### XVII.—IN CAIRO AND OUT OF IT.

### Hotels.

For those to whom expense is "no object," the choice of hotels in Cairo is large. There is a kind of historic interest attaching itself to Shepheard's which makes the lover of romance choose that excellent establishment in preference to all others, but there are plenty from which to select, all comfortable and well managed, and all somewhat expensive. If health is your object in visiting Egypt it is certainly desirable to live outside the town. Luxor and Assouan are far preferable to a large city like Cairo, but if you wish to have all the pleasures and gaieties of town and the advantages of the purest air, you can combine everything at the Mena House Hotel, eight miles from Cairo. A residence at this delightful house may be managed at pension terms, starting from 12s. per day. Two coaches run daily to the city, and there is no lack of interest or agreeable society in the hotel. It is close to the Pyramids and the Sphinx, and has in all ways much to recommend it. To those who like to study the idiosyncrasies of their fellow creatures there is a field for observation in the play of life and character displayed in the ever-changing groups of tourists, scientists, and general visitors who arrive and depart daily in pursuit of the immovable stone wonders so close to Mena House.

### Luxor.

This has for many years been a favourite resort for those with lung trouble. It is considerably warmer than Cairo, but the heat is so much tempered by the delicious dryness and clearness of the atmosphere that it is not felt to be in the least oppressive. Residence here is very charming; only to be alive in this delicious climate is a joy. The Grand Hotel Thewfikieh is very reasonable; pension from 12s. a day. There is a resident English physician and a nurse at Luxor and also at Assouan. The inexhaustible sources of interest in the antiquities in and around Luxor, and the celebrities of the scientific and social world who flock to visit them, prevent residence from ever becoming dull or prosaic here.

### Assouan.

Still further south lies Assouan, a trifle more bracing than Luxor, though warmer. The hotel is good but dearer than at Luxor, on account of its greater distance from the centres of civilisation. The near neighbourhood of the First Cataract adds a charm to residence here, and also the ability for the healthy to explore up to Wadi Halfa and the Second Cataract adds great interest. I shall speak of the steamer excursions later on.

### Cairo Itself.

It is unnecessary to have a guide whose presence usually runs up the price of everything in the bazaars, but it is equally unadvisable for ladies to explore the old quarters and bazaars alone. There is no danger, but the crowd in parts is so dense that it is a matter of some difficulty to get along. Try to secure the escort of some fellow countryman who knows the ropes. If you attempt to bargain with the natives you will come off like a chicken newly plucked, and probably have the mortification to find you have purchased Oriental embroideries and inlaid work made in Birmingham. The best day for the bazaars is Thursday. It is also necessary to employ the tactics described by King Solomon with regard to commercial transactions in these places, the buyer must continually cry "It is nought!" Depreciate generally the articles tendered, steadfastly refuse the price demanded, and resolutely turn to depart; this move, if executed with determination, generally brings an Oriental to reason.

### The Ascent of the Pyramids.

I cannot say I recommend this gymnastic feat to ladies; perhaps because I have not done it myself my feelings may partake of those of the fox who had lost his tail! To watch the start and observe the effect of the frightful fatigue afterwards was enough for me. If, however, ladies mean to attack the ascent they must be suitably dressed, that is with loose,

The Pyramids at Rest.

easy bodices, knickerbockers, and a very short skirt, with no braid or trimmings that can by any means catch on projections. It is an undertaking only fit for the exceptionally strong and athletic. When you reflect that the ascent is made by continuous steps of three feet in height—a tremendous stride unless you are of unusual height—and that the Arabs tug you up by the arms till you feel they must certainly give way at the shoulders, and that these hardened sinners allow you no breathing space between the horrible leaps and bounds—I leave you to judge whether you feel up to it. If you venture, by all means engage a third ruffian to "shove" (no other word describes the process) behind, which somewhat mitigates the suffering, and insist upon periodical rests. The whole charge, including everything, is from 12 to 14 piastres. The interior of the Great Pyramid is stuffy beyond description, reeking with the smell of thousands of bats. I did not venture far. Indeed, it is not very wholesome; the lack of pure air gives one an inclination to faintness. The Sphinx must not be attempted the same day, but should you be staying at Mena House you will be living in companionship with these wonders, and can take your own time in enjoying them. Nothing is more exquisitely beautiful and striking than a moonlight visit to the Pyramids and the Sphinx. Even without them moonlight in Egypt is a thing never to be forgotten or surpassed. Next week I shall hope to tell you something of the expenses (by no means small), and the arrangements necessary, for excursions up the Nile.

### TRAVEL NOTES AND QUERIES.

La Bourboule (Evening Star).—First-class fare, viâ Dover and Calais, £5 0s. 10d.; viâ Folkestone and Boulogne, £4 15s. 11d. A cheaper way considerably is by Southampton and Havre, £3 17s. 6d. The journey to Mont Dore is the same sum. The season lasts from June to September. Your doctor will tell you which is the more suitable for your friend.

A Sketching Tour (Pen and Ink).—As you say you can use water colour or oil equally well, my advice would be certainly the former. The appliances for oil sketching are heavy to carry, which is of importance when you have to pay for all your luggage, and sitting in cramped places, such as doorways or insecure ledges of rock, disaster to one's clothes has to be apprehended from the upsetting of dippers, falling of brushes, &c.

Algiers (Magic).—The hotels cannot be called cheap exactly, but they are far more reasonable than those in Cairo or on the Riviera. You would, of course, choose one in Mustapha Superieure, not in the town itself, for a lengthened sojourn. Prices range from 12 fr. 50 upwards. If you are a good French scholar, and understand the art of bargaining, you might find rooms, but I think the economy is very doubtful.

*(For Travel Advertisements see Page xvi.)*

### Notes and Queries.

The contents of the Editor's Letter-box have now reached such unwieldy proportions that it has become necessary to establish a hard and fast rule regarding Answers to Correspondents. In future, all questions requiring replies will continue to be answered in this column without any fee. If an answer is required by letter, a fee of half-a-crown must be enclosed with the note containing the enquiry. We are always pleased to help our numerous correspondents to the fullest extent, and we can trust them to sympathise in the overwhelming amount of writing which makes the new rules a necessity.

Every communication must be accompanied by the writer's name and address, otherwise it will receive no attention.

*Percentage on Fees.*

(11) I should feel greatly obliged if you can tell me if it is usual for nurses working on the co-operative system to be charged percentage on the quarantine fee.—*Sister.*

Yes; a percentage is charged on all the takings of the nurse.

*Inflamed Gums.*

(12) Could you tell me if a case of very sore and inflamed gums is one for a doctor or a dentist?—*F. G. J.*

Consult a surgeon-dentist.

*Poison Bottles.*

(13) Would it be possible to get bottles for poisons, so that the use for which they are intended could be detected by the touch? 2. Are there any firms to which we could apply for samples?—*Nurse Alma.*

Bottles of unusual shape, marked by irregular surfaces, are now constantly used for poisons. Samples would be supplied on application by most firms of makers. The great difficulty is that people will not throw away their old bottles, and thus it happens that one constantly finds old "poison" bottles used for ordinary purposes, and thus carelessness is induced. "Poison" bottles should always be destroyed when done with.

*Sulphur Baths.*

(14) Will you kindly inform me where I could obtain "sulphur baths" for a patient in London?—*Baths.*

Medicated baths of all kinds may be obtained at University College Hospital. Apply to the Secretary.

*Corsets.*

(15) Could you kindly tell me of a good invalid corset-maker, as near Cavendish Square as possible?—*A. S.*

We believe that the nearest to Cavendish Square are Messrs. Josephine et Cie., Regent Street.

*Outdoor Uniform.*

(16) Can you tell me if it is the case that the matrons of many of the best-known hospitals refuse to allow their nurses to wear uniform out-of-doors? The large London hospital to which I have just been admitted as a candidate does not permit any nurse belonging to it to appear out-of-doors in uniform. They must all change into ordinary walking dress before going out; in fact there is no outdoor uniform at all belonging to the hospital. This seems rather a pity. I do not know whether members of the nursing profession would agree with me; I should be glad to hear through your columns if any do so or not. Can you tell me the reason of this regulation?—*A. E.*

The wearing or not of uniform is entirely a matter of the regulations of individual institutions. The reason why so many discountenance the wearing of outdoor uniform is that the dress is not now distinctive of a trained nurse, and is therefore becoming discredited in the eyes of the public and no longer affords protection to the wearer. At the same time, when hours for exercise are short, we think the undoubted convenience of being able to save time by merely putting on a cloak instead of "changing" when going out for a walk ought to be considered by the authorities. The wearing of a uniform when "off duty" for a longer period is quite another matter.

*An Antidote to Snake-bite.*

(17) We thank a correspondent for kindly sending us the following reply to Query 237: "Hag-worm" is the local name in the English Lake District for the adder. "First aid" may be rendered by sucking the wound, and applying a ligature above it to prevent the poison flowing on in the blood. Give stimulants if there is faintness. It should be shown to a doctor.

## Figures and Tables

**Figure f1:**